# Cerium Oxide Nanorods Synthesized by *Dalbergia sissoo* Extract for Antioxidant, Cytotoxicity, and Photocatalytic Applications

**DOI:** 10.3390/molecules27238188

**Published:** 2022-11-24

**Authors:** Mir Waqas Alam, Sumaira Naeem, Sheikh Muhammad Usman, Qudsia Kanwal, Amal BaQais, Fatimah Saeed Aldughaylibi, Insha Nahvi, Noushi Zaidi

**Affiliations:** 1 Al Bilad Bank Scholarly Chair for Food Security in Saudi Arabia, The Deanship of Scientific Research, The Vice Presidency for Graduate Studies and Scientific Research, King Faisal University, Al-Ahsa 31982, Saudi Arabia; 2 Department of Physics, College of Science, King Faisal University, Al-Ahsa 31982, Saudi Arabia; 3 Department of Chemistry, University of Gujrat, Gujrat 50700, Pakistan; 4 Hunza Sugar Mills Private Limited (Distillery Division), Lahore 54000, Pakistan; 5 Department of Chemistry, The University of Lahore, Lahore 54000, Pakistan; 6 Department of Chemistry, College of Science, Princess Nourah Bint Abdulrahman University, Riyadh 11671, Saudi Arabia; 7 Department of Basic Sciences, Preparatory Year Deanship, King Faisal University, Al-Ahsa 31982, Saudi Arabia

**Keywords:** cerium oxide, nanorods, response surface methodology, water purification, methyl orange, photocatalysts

## Abstract

In this study, cerium oxide nanorods (CeO_2_-NRs) were synthesized by using the phytochemicals present in the *Dalbergia sissoo* extract. The physiochemical characteristics of the as-prepared CeO_2_-NRs were investigated by using ultraviolet-visible spectroscopy (UV-VIS), scanning electron microscopy (SEM), Fourier transform infrared spectroscopy (FTIR), and X-ray diffraction analysis (XRD). The SEM and UV-VIS analyses revealed that the acquired nanomaterials possessed a rod-like morphology while the XRD results further confirmed that the synthesized NRs exhibited a cubic crystal lattice system. The antioxidant capacity of the synthesized CeO_2_-NRs was investigated by using several in vitro biochemical assays. It was observed that the synthesized NRs exhibited better antioxidant potential in comparison to the industrial antioxidant of the butylated hydroxyanisole (BHA) in 1,1-diphenyl-2-picrylhydrazyl (DPPH) assay. The biochemical assays, including lipid peroxidation (LPO), total antioxidant capacity (TAC), and catalase activity (CAT), were also performed in the human lymphocytes incubated with the CeO_2_-NRs to investigate the impact of the NRs on these oxidative biomarkers. Enhanced reductive capabilities were observed in all the assays, revealing that the NRs possess excellent antioxidant properties. Moreover, the cytotoxic potential of the CeO_2_-NRs was also investigated with the MTT assay. The CeO_2_-NRs were found to effectively kill off the cancerous cells (MCF-7 human breast cancer cell line), further indicating that the synthesized NRs exhibit anticancer potential as well. One of the major applications studied for the prepared CeO_2_-NRs was performing the statistical optimization of the photocatalytic degradation reaction of the methyl orange (MO) dye. The reaction was optimized by using the technique of response surface methodology (RSM). This advanced approach facilitates the development of the predictive model on the basis of central composite design (CCD) for this degradation reaction. The maximum degradation of 99.31% was achieved at the experimental optimized conditions, which corresponded rather well with the predicted percentage degradation values of 99.58%. These results indicate that the developed predictive model can effectively explain the performed experimental reaction. To conclude, the CeO_2_-NRs exhibited excellent results for multiple applications.

## 1. Introduction

Nanomaterials (NMs), materials possessing any one dimension confined within the nanometer regime (1–100 nm), have been extensively utilized in recent years for numerous applications including catalysis [1], energy conversion and storage [2], medical [3], and electronic applications [4], etc. The NMs particularly exhibit extraordinary potential in therapeutics, owing to their exceptionally unique physiochemical characteristics in comparison to their bulk counterparts. These unique characteristics are imparted owing to the smaller size of these NMs (i.e., in nanometer range). Most of the applied research associated with NMs depends heavily on the production of NMs with variable sizes and shapes with the surface of the NMs specifically tailored with the tunable moieties for specific applications [5]. However, the synthetic methodologies utilized for the development of NMs are rather complicated and require multiple instruments, hazardous chemicals, and harsh working environments, which is not preferable in accordance with the recent interest shown by the scientific community toward green chemistry [6]. For instance, Manjula et al. [7] documented the combination of co-precipitation and calcination methodologies for the synthesis of the cerium oxide (CeO_2_) nanorods (NRs) with the requirement of a high temperature of 450 °C sustained for four hours for the acquisition of the NRs’ morphology. Kim et al. [8] documented the chemical methodology for the preparation of the CeO_2_-NMs and specified that acquiring the specific morphology of sphere (nanoparticles) or rod requires very careful manipulation of the reaction conditions. The authors utilized the hazardous solvents (including ammonia and ammonium hydroxide) and a high temperature of 300 °C for the preparation of CeO_2_-NRs. These studies highlighted the need to develop new greener synthetic methodologies for the synthesis of morphologically specific NMs.

Cerium (Ce) is a well-known rare earth metal associated with the lanthanide series in the periodic table. In terms of oxidation states, cerium exhibits either Ce^4+^ or Ce^3+^ oxidation states depending on the reducing/oxidizing microenvironments owing to its reversible binding with oxygen [9]. The subsequent loss of the oxygen atom or the reduction capability of Ce^4+^ into Ce^3+^ imparts the antioxidant property to the synthesized CeO_2_-NMs, which makes it an exceptional material to be investigated for numerous medical applications [10]. This oxygen vacancy observed in CeO_2_ has largely been utilized for the biomedical applications but the complicated synthetic methodologies of the CeO_2_-NMs have always been seen as one of its major limitations that reduces the practical applicability of these materials [11].

Keeping the significance of the CeO_2_-NMs in mind, this study is designed to develop the morphology-specific (i.e., rod-shaped) CeO_2_-NMs by utilizing the greener approach with moderate temperature conditions. The biomedical and catalytic applications of the NMs will also be tested to ensure that the biomedical potential of the CeO_2_-NMs is not lost by using the proposed methodology. Our research group recently addressed the same issue for Cadmium oxide (CdO) and synthesized the cauliflower-shaped CdO nanoflowers under ambient conditions by utilizing a concentrated extract of *Dalbergia sissoo* fresh leaves [12]. *Dalbergia sissoo* is one of the most well-known traditional Pakistani plants utilized as herbal medicine by local communities for addressing numerous ailments. The selection of this plant for the synthesis of the CeO_2_-NRs was based on the fact that our research group is well familiar with the phytochemical aspects of this plant. The phytochemical profiling (with the major phytochemicals identified as sissooic acid, quercetin-3-O-rutinoside, biochanin A, kaempferol-3-O-rutinoside, and dalsissoside) along with the cytotoxicity studies mutagenicity potential, anti-epileptic activity, antioxidant/reduction potential, and antimicrobial potential of the *D. sissoo* had already been previously investigated by our group [13,14,15]. Furthermore, the NMs of magnesium oxide (MgO) along with CdO were also synthesized by using the *D. sissoo* extract for photocatalytic applications by our research group [12,15]. In this study, an attempt was made to utilize the same strategy to engineer the rod-shape CeO_2_-NMs by using the *D. sissoo* extract and the synthesized NRs, which will be utilized to address numerous medical and catalytic applications.

The production of reactive oxidative groups as byproducts of (normal/pathological) human metabolic activities is regarded as a naturally occurring phenomenon. Recent research has linked the overproduction of these reactive species to a variety of serious health problems, including cardiovascular diseases, weakened immune systems, neurological diseases, the onset of cancer, and other degenerative diseases [16]. Every living organism is equipped with some type of defense mechanism to counteract these potential oxidative stresses. [17]. Similarly, synthetic antioxidants such as butylated hydroxyanisole (BHA) and butylated hydroxytoluene (BHT) are used as food additives to prevent oxidative decomposition. However, nutritionists are somewhat concerned about the safety issues surrounding the use of these compounds, and the focus has shifted toward identifying natural anti-oxidants and nutraceuticals to improve the nutritional quality of consumers [16]. Consequently, the development of new antioxidant materials that can be used in place of these harmful synthetic antioxidants is of the utmost importance.

Another problem that has attracted the interest of the scientific community in recent years is the presence of noxious pollutants in aqueous environments, as these pollutants ultimately end up directly/indirectly impacting human beings. Numerous removal methodologies (including adsorption [16], catalytic reduction, Fenton oxidation, flocculation, coagulation, etc.) have been utilized to remove these pollutants from water reservoirs. However, these methodologies suffer from the drawbacks of incomplete removal, economically unviable, requirement of additional setups, and extensive recovery procedures [17]. Here, we have utilized the process of photocatalysis for the removal of the noxious pollutant of methyl orange (MO) by using the synthesized CeO_2_-NRs. The complete removal/mineralization of the pollutant into less harmful products of water (H_2_O) and carbon dioxide (CO_2_), with economical time consumption, and high removal rate are some of the major advantages of photocatalysis over other conventional techniques [18]. The NRs were prepared by using the phytochemicals present in the *D. sissoo* extract as the stabilization and fabrication medium. The synthesized CeO_2_-NRs were characterized by using different analytical techniques. Furthermore, the antioxidant, antibacterial, and photocatalytic potential of the synthesized CeO_2_ was also investigated in this study.

## 2. Materials and Methods

### 2.1. Materials

The chemical reagents including extra pure cerium nitrate hexahydrate ([Ce(NO_3_)_3_.6H_2_O]; 99.999%), butylated hydroxyanisole (BHA; ≥98.5%), malondialdehyde (MDA; ≥96%), 2-thiobarbituric acid (TBA; ≥98%), methyl orange (MO; 85%), trichloroacetic acid (TCA; ≥99%), 2,2-diphenyl-1-picrylhydrazyl (DPPH), 2, 4, 6-tripyridyl-s-tiazine (TPTZ; ≥98%), human breast cancer cell line (MCF-7), phosphate-buffered saline (PBS, pH = 7.4), hydrogen peroxide (H_2_O_2_; 35%), dimethyl sulfoxide (DMSO), and n-butanol were purchased from Sigma-Aldrich, St. Louis, MO USA.

### 2.2. Preparation of D. sissoo-Stabilized CeO_2_-NRs

For the preparation of the *D. sissoo* bioextract, fresh leaves of *D. sissoo* were collected from the botanical garden of the University of Gujrat. The collected leaves were properly cleaned, washed, dried, and ground into fine powder for further experimentation. A total of 10 g of the powdered *D. sissoo* leaves was added to 150 mL of the double-distillated water and was continuously stirred for 24 h. The acquired extract was filtered and the filtrate was stored in air-tight sample bottles at 4 °C. For the preparation of the CeO_2_-NRs, 10 mL of 3 mM of Ce(NO_3_)_3_.6H_2_O was added to 100 mL of *D. sissoo* extract. The reaction mixture was continuously stirred for 6 h at the temperature of 75 ± 3 °C until white precipitates were obtained. The formed precipitates were separated by using centrifugation and washed with water and ethanol several times to remove any impurity present in the sample. The precipitates were further placed in an oven for 2 h at a temperature of 150 °C. The acquired CeO_2_-NRs were stored in air-tight sample bottles for further experimentation [19].

### 2.3. Characterization

The ultraviolet-visible (UV-VIS) spectroscopy of the synthesized CeO2-NRs was carried out by using double-beam PerkinElmer, Incorporation® (LAMBDA-365+; Waltham Massachusetts, USA) spectrophotometer. The Bruker Corporation (Aubrey, Texas, USA) diffractometer was utilized for performing the X-ray diffraction (XRD) analysis. The LYRA-3 (TESCAN; Edgerton, Missouri, USA) microscope was used for identifying the morphological characteristics of the synthesized CeO2-NRs via scanning electron microscopy (SEM). The Fourier transform infrared spectroscopy (FTIR, Carry-630 Agilent; Santa Clara, California, USA), in the range of 4000–400 cm−1, was also performed for identifying the functional group of the phytochemicals involved in the stabilization of the CeO_2_-NRs.

### 2.4. DPPH Radical Scavenging Assay

The DPPH radical scavenging assay was used for investigating the antioxidant potential of CeO_2_-NRs. In brief, the solution containing the 23 mg/mL solution of DPPH in ethanol was prepared and subsequent absorbance of the DPPH radical ion was measured at 517 nm. The BHA was used as the positive control for the test. All the tests were replicated thrice to ensure the precision of the acquired results. The prevention capacity of the active radical was measured by using the standard of ascorbic acid [20].

### 2.5. Biochemical Assays for Human Lymphocytes Incubated with CeO_2_-NRs

The human blood sample (2 mL), from a healthy donor, was collected and centrifuged at 3000 rpm for 15 min. The acquired white-ish layer (that was formed just below the upper plasma layer) was removed and collected in a separate sample bottle. The collected lymphocytes were washed thrice with ammonium chloride to remove any impurity (particularly red blood cells) and the material was suspended in PBS to be stored at 4 °C [21]. The acquired lymphocytes were incubated with the synthesized CeO_2_-NRs in varying concentrations of 15, 30, 60, and 120 μmol/mL. The treated lymphocytes were tested for numerous oxidative stress biomarkers.

The total antioxidant assay (TAC) for the CeO_2_-NRs was investigated by using the ferric reducing ability of plasma (FRAP) methodology. This methodology determines the capability of the plasma (incubated with CeO_2_-NRs) to reduce Fe^3+^ ions into Fe^2+^ ions. The reduced Fe^2+^ ions exhibited a blue color complex with the TPTZ whose amount can be spectroscopically measured owing to the maximum absorbance of this complex observed to be at 593 nm [22].

The lipid peroxidation (LPO) methodology was also utilized to determine the antioxidant capacity of the lymphocytes incubated with CeO_2_-NRs. The extent of MDA produced during the acid heating reaction serves as the main species utilized for spectroscopically measuring the antioxidant activity. In a typical assay, 10 μL of lymphocyte samples containing CeO_2_-NRs in varying concentrations or a standard MAD solution and 40 μL of PBS were added to an Eppendorf tube placed in an ice bath. In each sample tube, TBA reagent (containing 100 μL of TBA, 30 μL of phosphotungstic acid, 50 μL of sodium dodecyl sulphate, and 200 μL of HCl) was added and these air-tight tubes were boiled for 25 min at 100 °C in the water bath. Next, 400 μL of n-butanol was added in the sample and the tubes were centrifuged at 3000 rpm for 10 min. The separated supernatants were added in a 96-well plate. The excitation/emission fluorescence wavelengths of 515/555 nm were measured using the microplate reader [23].

The catalase activity (CAT) was also assessed by observing the decrease in the value of the absorption intensity at 240 nm associated with H_2_O_2_ owing to the presence of the lymphocyte medium containing the CeO_2_-NRs. The specific CAT activity was measured in units/mL with one unit of enzyme equal to the enzyme required for consuming1 mol of H_2_O_2_ per minute [24].

### 2.6. Cytotoxicity of CeO_2_-NRs

The cytotoxicity potential of the CeO_2_-NRs was investigated with the MTT assay. This quantitative colorimetric evaluation was used to observe the activity of the enzymes. The MCF-7 (human breast cancer cell line) was seeded in a 96-well plate (with the density of 10,000 cells/well) and these plates were kept at 37 °C for 24 h. The CeO_2_-NRs (in varying concentrations of 0, 25, 50, 75, 100, and 250 μg/mL) were inoculated into the grown cell with 100 μL of the medium. After every 24 h of incubation, 5 mg/mL (20 μL) MTT dissolved in the buffer was added into every well. After incubation, the formazan crystals were secured while the remaining media were discarded. The crystals (shaped via MTT metabolism) were liquefied and dissolved into 100 μL of DMSO. The optical absorbance of the acquired plates was measured at 590 nm [25].

### 2.7. Photocatalytic Efficacy of CeO_2_-NRs

The photocatalytic potential of the CeO_2_-NRs was calculated by carrying out the model degradation reaction of MO. All the photocatalytic reactions were carried out under a 10 W ultraviolet (UV) lamp. The response surface methodology (RSM) was utilized as a means to develop the design of the experiment for this photocatalytic reaction. The central composite design (CCD) was developed by using the three variables of MO dose, photocatalyst dose, and pH while the parameter of the percentage degradation (*D*%) was used as the main response of the CCD. Twenty experiments were performed and the details of these experiments are provided in the Supplementary File. In a typical photocatalytic reaction, the specific amount of MO and photocatalysts (as specified by the design of the experiment CCD model) was added in a photocatalyst reactor. The reaction was left to be homogenized for 30 min to ensure the presence of adsorption/de-sorption equilibrium. The reaction mixture was then placed under a UV lamp for the progression of the reaction. A total of 3 mL of the reaction mixture was collected after regular intervals for measuring the absorbance (at a wavelength of 463 nm) for calculating the main response of D% as given by Equation 1.

(1) $$\;D\%=\frac{C_{0}-C_{res}}{C_{o}}\times 100\;\;\;\;\;\;\;\;\;\;\;\;\;$$

*C_o_* and *C_res_* represent the concentration of MO at the start of the photocatalytic reaction and at any time (t), respectively.

## 3. Results and Discussion

### 3.1. Synthesis

**Figure 1:**
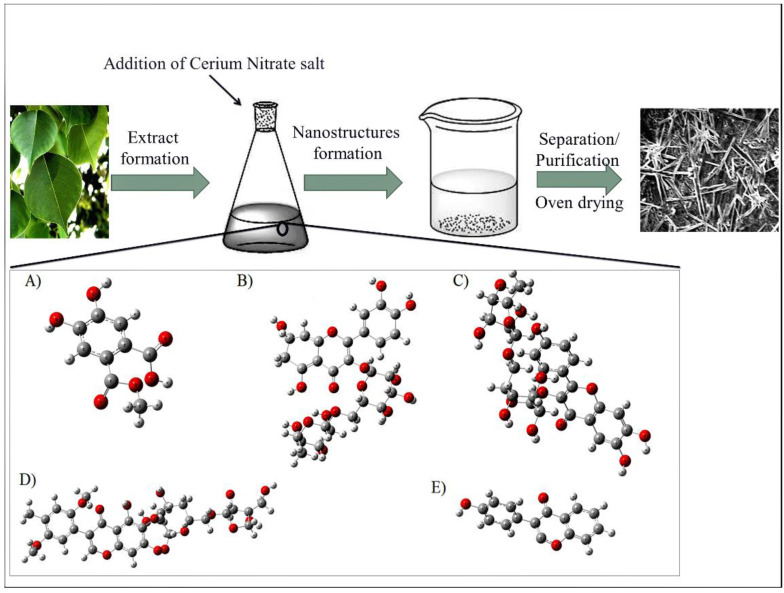
Synthetic scheme for the production of CeO_2_-NRs by using *Dalbergia sisoo* extract, here: (**A**) Sissoic acid; (**B**) Quercetin-3-O-rutinoside, (**C**) kaempferol-3-O-rutinoside, (**D**) Dalsissoside, and (**E**) Biochanin.

The phytochemicals present in the *D. sissoo* extract were utilized as fabricating as well as stabilizing agents for the production of CeO_2_-NRs. The physiochemical profiling detailing the identification of the phytochemicals involved has already been reported by our research group [14]. The extract was found to essentially contain numerous natural oxidants and reductants including tannis, reducing sugars, terpenoids, glycosides, proteins, and saponins [14]. In another work, we identified the following specific biomolecules including sissoic acid, biochanin A, quercetin-3-O-rutinoside, kaempferol-3-O-rutinoside, and dalsissoside which were present in abundance in the *D. sissoo* extract. The most significant biomolecule was found to be quercetin, which was observed in the UV-VIS spectrum of the *D. sissoo* extract [12,15]. The cumulative interaction of the phytochemicals associated with the precursor salt of the cerium was found to be responsible for the preparation of CeO_2_-NRs as indicated in Figure 1.

### 3.2. Characterization

**Figure 2:**
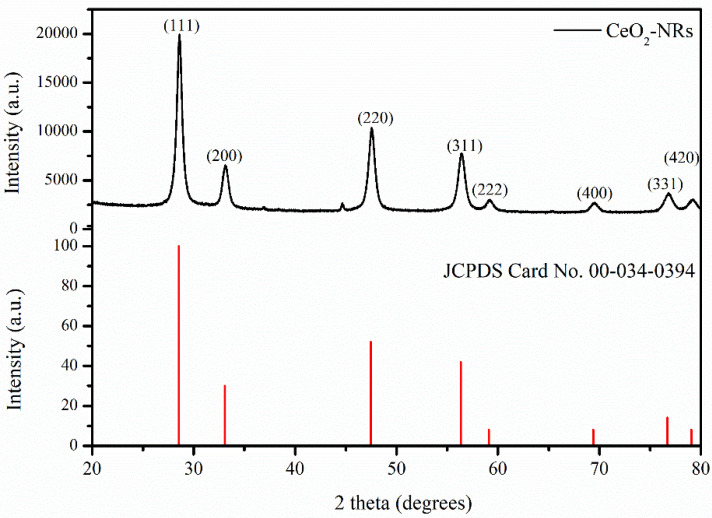
XRD spectrum of CeO_2_-NRs and the standard JCPDS Card no. 00-034-0394.

The successful fabrication of CeO_2_-NRs was primarily validated by using the XRD technique, as shown in Figure 2. The XRD spectrum indicated that the acquired sample possessed the cubic crystal lattice system with the observed lattice parameters of *a* = *b* = *c* = 5.4113 Å. The indexing of the XRD spectrum revealed it to have diffraction peaks at the 2θ values of 28.55 °, 33.08 °, 47.47. °, 56.33 °, 59.08 °, 69.40 °, 76.70 °, and 79.07 ° associated with the diffraction planes of (111), (200), (220), (311), (222), (400), (331), and (420), respectively. The XRD pattern correlated well with the standard CeO_2_-NRs JPCDS Card no. 00-034-0394. It was also found that the CeO_2_-NRs sample was extremely pure as no unindexed peak was observed in the case of the CeO_2_-NRs’ XRD spectrum [26].

**Figure 3:**
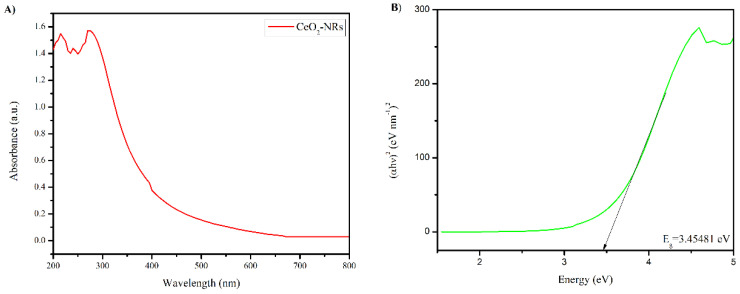
(**A**) UV-VIS spectrum of CeO_2_-NRs and (**B**) the Tauc plot for the band gap (*E_g_*).

The UV-VIS spectrum and the Tauc plot of the CeO_2_-NRs is presented in Figure 3. As indicated in Figure 3A, the CeO_2_-NRs do not exhibit the optical absorbance band in the visible region. The characteristics band at 285 nm of the CeO_2_-NRs was observed in the ultraviolet (UV) region, indicating that the UV lamp should be preferred for the activation of the CeO_2_-NRs for photocatalytic applications [25]. The Tauc equation (as presented in Equation 2) was also utilized to determine the band gap (*E_g_*) values of the synthesized NRs.

(2) $$\alpha hv=A{\left(hv-E_{g}\right)}^{2}\;\;\;\;\;\;\;\;\;\;\;\;\;\;\;\;\;$$

where *hv* represents the energy of the incident photon, *h* exhibits the Planck’s constant, α is the optical coefficient, *v* represents the frequency of the photon, and *A* represents the transition constant. The direct *E_g_* value of the CeO_2_-NRs was found to be 3.4541 eV, which is again indicative of the fact that the engineered NMs are capable of utilizing the UV region effectively [27].

**Figure 4:**
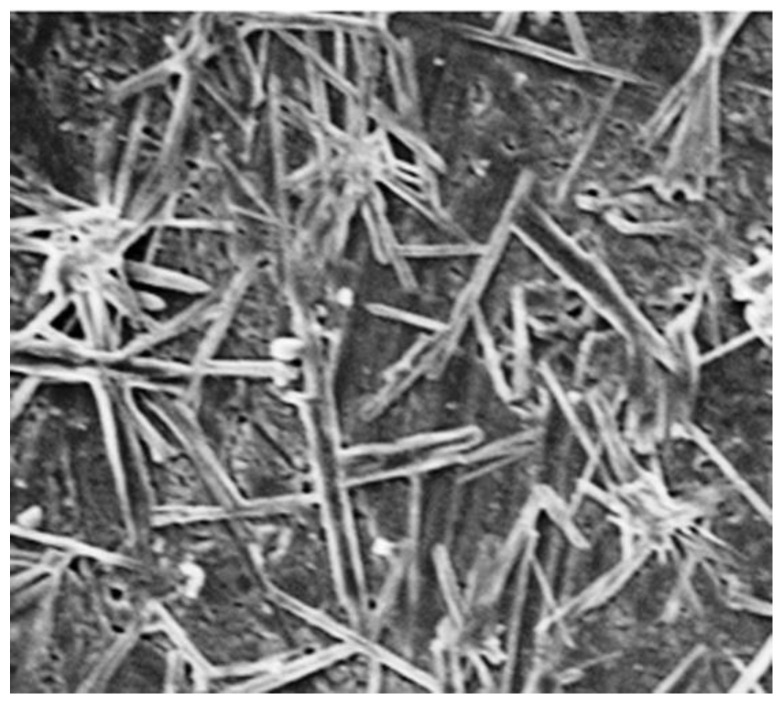
SEM micrographs of CeO_2_-NRs.

The morphological characteristics of the CeO_2_-NRs were studied by using SEM as presented in Figure 4. The SEM micrograph confirms that the engineered material exhibited rod-like morphology. However, the size of the synthesized CeO_2_-NRs was not uniform, which is in accordance with the preparation methodology as the green phytochemical-based synthetic methodology is a crude technique for the preparation of NMs. The rough surface observed in the case of the CeO_2_-NRs further suggests that the synthesized NRs will be effective for surface-based applications.

**Figure 5:**
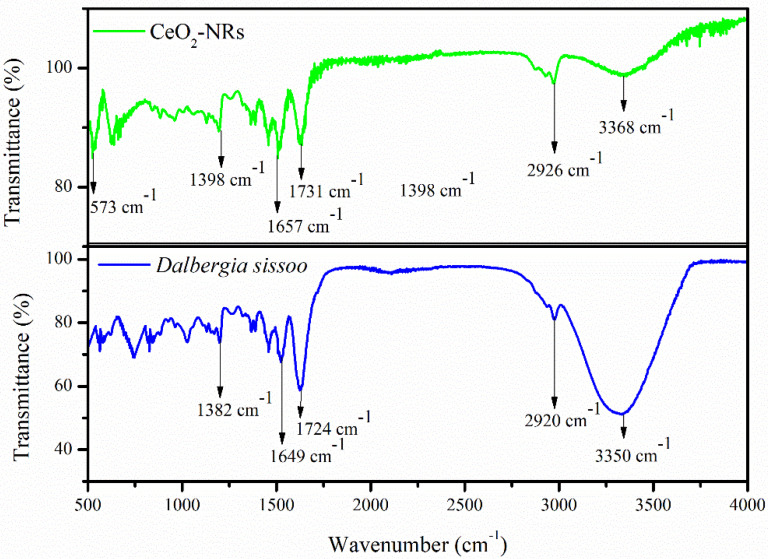
FTIR spectrum of *D. sissoo* and CeO_2_-NRs.

The FTIR spectrum of the *D. sissoo* extract and the CeO_2_-NRs is elaborated in Figure 5. The presence of all the major characteristic peaks of the *D. sissoo* extract in the FTIR spectrum of the CeO_2_-NRs reveals that the phytochemicals of the *D. sissoo* extract act as a stabilization medium for the fabricated NRs. The presence of a strong band at 3350 cm^−1^ represents the vibrational frequency of the hydroxyl group, indicating that the *D. sissoo* extract is predominantly composed of polyphenols. This band was reduced in intensity in the case of the CeO_2_-NRs, which is indicative of the fact that these groups strongly interacted with the CeO_2_-NRs, resulting in the displacement of this peak from 3350 cm^−1^ to 3368 cm^−1^ [28]. The decrease in the intensity can be further associated with the process of oven drying, as high temperatures also removed any attached water from the CeO_2_-NRs and the remaining band only indicated that the polyphenols of the phytochemicals adhered to the surface of the NRs as the stabilization medium [12]. The decrease in the intensity can be further associated with the process of oven drying as high temperatures also removed any attached water from the CeO_2_-NRs. The subsequent bands at 2920 cm^−1^ (CH group; stretching), 1724 cm^−1^ (C=O group), 1649 cm^−1^ (C=C group), and 1382 cm^−1^ (CH group; bending) were present in the *D. sissoo* extract. These bands were slightly displaced in the case of the CeO_2_-NRs, indicating that the phytochemicals indeed interacted with the CeO_2_-NRs [29]. The presence of the characteristics additional band at 573 cm^−1^ associated with the vibrational frequency of the Ce-O bond further validates the formation of CeO_2_-NRs [29].

### 3.3. DPPH Assay for CeO_2_-NRs

**Figure 6:**
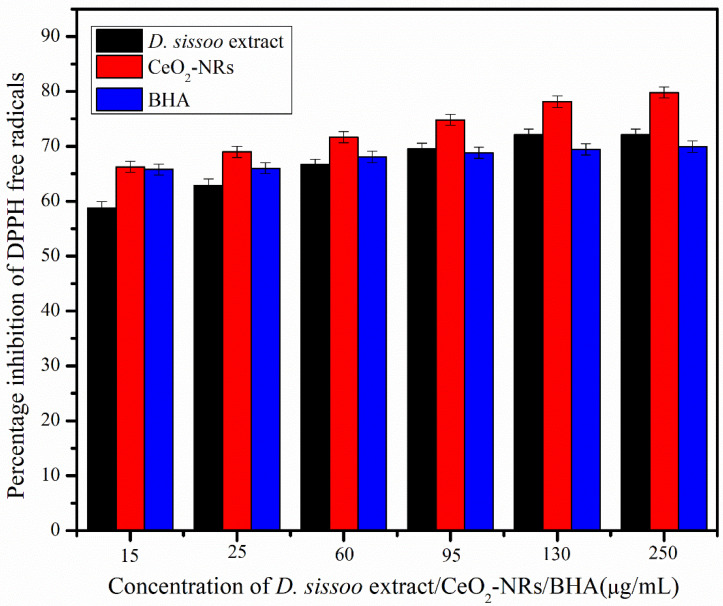
The DPPH assay for *D. sissoo*, CeO_2_-NRs, and BHA.

The antioxidant capacity of the D. sissoo extract and the CeO_2_-NRs was tested by using the DPPH assay as indicated in Figure 6. It was observed that the CeO_2_-NRs exhibited even better antioxidant results in comparison to the industrial antioxidant of BHA at all the concentrations. Moreover, the observed antioxidant activity of the CeO_2_-NRs was found to be dose-dependent, as with the increase in concentration from the series 1 (15 μg/mL) to series 6 (250 μg/mL), the percentage inhibition of the DPPH free radicals by CeO_2_-NRs also increases. This is to be expected as CeO_2_ (apart from having the antioxidant potential of the phytochemicals present on the surface) also possesses lower ratios of Ce^3+^/Ce^4+^ on the surface that contribute to enhance its antioxidant potential in comparison to the extract. Dutta et al. [30] documented similar results for the CeO_2_-NRs. Furthermore, it should also be observed that the *D. sissoo* extract itself acted as a rather good antioxidant material and surpassed the antioxidant efficacy of the BHA at the higher concentrations. At lower concentrations of the extract, the BHA exhibited better results than the *D. sissoo* extract. This could be further interpreted as an interesting insight regarding the effectiveness of the *D. sissoo* extract, revealing that a concentrated extract is more effective for achieving better pharmacological applications of this traditional ethno-medicinal plant [31].

### 3.4. Biochemical Assays for Human Lymphocytes Incubated with CeO_2_-NRs

Table 1 provides the results of the biochemical assays documented for the human lymphocytes incubated with the varying concentrations of CeO_2_-NRs. The TAC assay revealed that the increasing concentration of CeO_2_-NRs in the lymphocytes enhanced the FRAP values. This is indicative of the fact that the CeO_2_-NRs possessed antioxidant potential and effectively facilitated the Fe^3+^ ions reduction into Fe^2+^ ions. The reduced Fe^2+^ ions then combined with the TPTZ to provide a complex which was spectroscopically measured. The enhanced concentration of the Fe^2+^ ions indicated that from the concentration of 15–60 μmol/mL, an increase in antioxidant potential with the concentration was observed, while at the concentration of 120 μmol/mL, the trend was reversed. This is crucial as it reveals that the lower concentrations of CeO_2_-NRs were found to be more effective in terms of antioxidant potential [32].

The significant decrease in the concentration of peroxide radical was observed in the case of the human lymphocytes incubated with the CeO_2_-NRs. The optimum concentration for this assay was also found to be 60 μmol/mL, which is again indicative of the fact that the antioxidant potential of the CeO_2_-NRs was effective at lower concentration of ROS [24]. Increasing the dose of the CeO_2_-NRs in the system also resulted in an increase in the peroxide radicals scavenging ability of the NRs as well [33].

### 3.5. Cytotoxicity Effects of CeO_2_-NRs on the MCF-7 Cell Line

**Figure 7:**
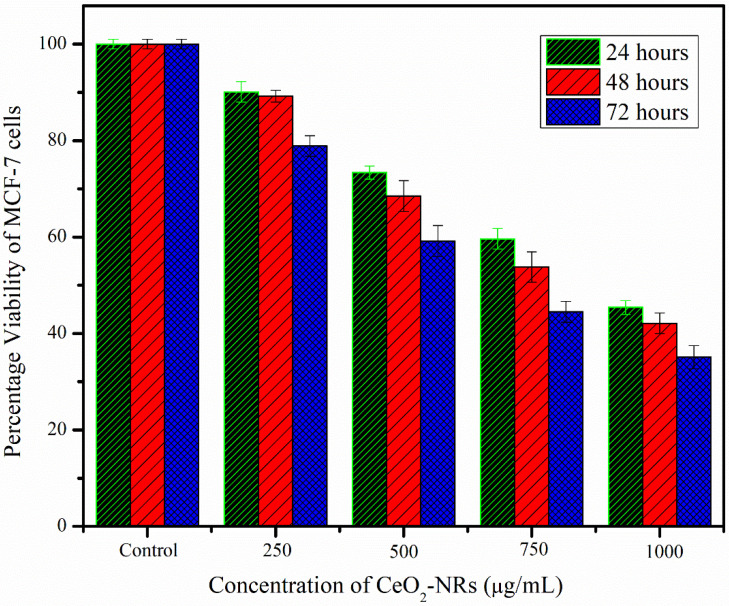
The cytotoxic potential of the synthesized CeO_2_-NRs against MCF-7 cells.

The cytotoxic effect of CeO_2_-NRs was investigated by using the human breast cancer MCF-7 cell line as indicated in Figure 7. It was observed that the addition of the CeO_2_-NRs significantly reduced the percentage viability of the MCF-7 cell line. This cytotoxic effect is attributed to the extremely small size of the NRs as the dangling bonds available at the surface of the NRs make them chemically reactive particularly for biochemical catalytic reactions [34]. These NMs can penetrate into the cells owing to their high chemical reactivity and cause the lysis of the cell. The acquired results correspond rather well with the findings of Navada et al. [35]. The decrease in the viability of the cancerous cells was also found to be dose- as well as time-dependent. The higher doses (i.e., 100 μg/mL) and the treatment time duration of 72 h were found to be most effective with a maximum decrease of 35.124% in the viability of the MCF-7 cells.

### 3.6. RSM-Based Optimization of Photocatalytic Activity of CeO_2_-NRs

#### 3.6.1. Assortment of Predictive Model

**Figure 8:**
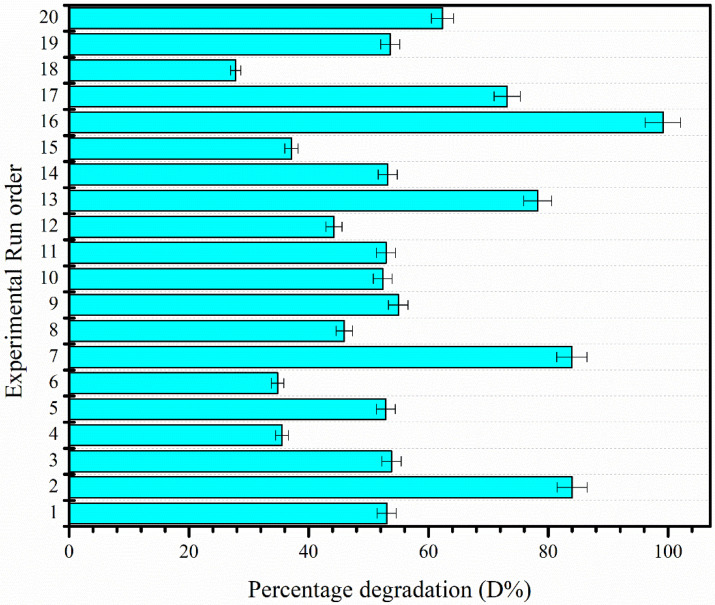
The CCD-based twenty photocatalytic reactions for the degradation of MO performed under the reaction conditions specified by the standard 3-factor RSM design of experiment.

The three-factor CCD matrix and the acquired photocatalytic results acquired for the photocatalytic degradation reaction of MO are presented in Figure 8. The detailed design of the experiment as well as the selection tests performed to identify the right model are presented in the supplementary information. The response factor (percentage degradation of MO) was found to be a second-order polynomial (quadratic model), as presented in Equation 3. The selection of the model was achieved by using Fischer’s exact test (*p*-value), the F-statistics test (F-value), regression square (R^2^), adjusted R^2^, and predicted R^2^ as detailed in the Supplementary File. The analysis of variance (ANOVA) test was also performed for further experimentation.

(3) $$\begin{array}{cc} Percentage\;degradation & \\ & =+53.29-13.70\;A+13.69\;B+6.30\;C-8.13\;AB\\ & -3.17\;AC-1.54\;BC+1.84\;A^{2}-0.4091B^{2}+3.59\;C^{2}\;\;\;\;\;\;\;\;\;\;\;\; \end{array}$$

where the factors A, B, and C represent the coded individual factors of MO dose, photocatalyst dose, and pH units, respectively. The factors of AB, AC, and BC represent the interaction terms while the remaining A^2^, B^2^, and C^2^ represent the squared terms.

#### 3.6.2. ANOVA

The ANOVA test was applied on the quadratic model to verify the validity of the selected model as explained in Table 2. The statistically significant p-value (i.e., <0.0001) indicated that the predictive model selected for explaining the photocatalytic degradation of MO correlated rather well with the observed experimental results. Furthermore, the individual terms of A, B, C, and the interaction term of AB were found to be statistically significant. The other interaction terms of AC and AD were not found to be statistically significant, indicating that the correlation among these factors did not impact the %D of the reaction as much. In the case of the ANOVA test, it was further observed that our predictive model provided the F-value of 149.5, which was found to be rather impactful. Conventionally, the model is considered significant when the tabulated F-value (15.11) is almost four or five times smaller in comparison to the calculated F-value [36]. Moreover, the p-value for the confidence interval of 99% and 95% (i.e., under the significance levels of 1% and 5%, respectively) is also an important way to assess the validity of the model. A p-value of less than 0.05 (for a confidence interval of 95%), or even better with a p-value of less than 0.01 (for a confidence interval of 99%) is typically considered statistically significant. The p-value for the elected model was found to be <0.0001, which indicated that the model is rather good and lies within the significance level of 1% [37].

Combining both the acquired model and ANOVA results revealed that the factors of A, B, C, and AB should be given special emphasis for modulating the percentage degradation of MO. The factor A (MO dose) had a negative sign in the model, indicating that any increment in the factor A will negatively impact the D%. The same results were observed in the case of factor AB. However, the value of the interaction coefficients of the individual terms A, B, and C was found to be rather higher in comparison to the interaction term of factor AB. This further indicated that the optimization of this reaction is heavily dependent on the individual factors rather than on the interaction terms [38].

#### 3.6.3. Diagnostics Plots for the Selected Model

**Figure 9:**
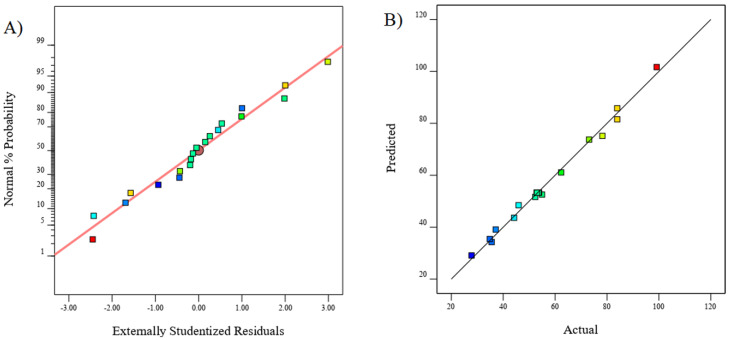
Diagnostics tests including (**A**) Normal probability plot and (**B**) Predicted vs. experimental D% values for the response of photocatalytic degradation of MO.

The validity of the selected quadratic model was further investigated by numerous validity plots, as indicated in Figure 9. A normal plot associated with the residuals of the photocatalytic degradation of MO is presented in Figure 8A. The slight deviation of the acquired experimental values from the straight line is expected as the errors can never be completely eradicated from the experiments [39]. The acquired response in terms of the D% values for the photocatalytic reaction of MO in the presence of the CeO_2_-NRs was also found to be rather comparable with the predicted values (Figure 8B), indicating that the selected model was significantly valid for explaining the behavior of this particular reaction [37].

#### 3.6.4. RSM-Based Three-Dimensional and Contour Plot

**Figure 10:**
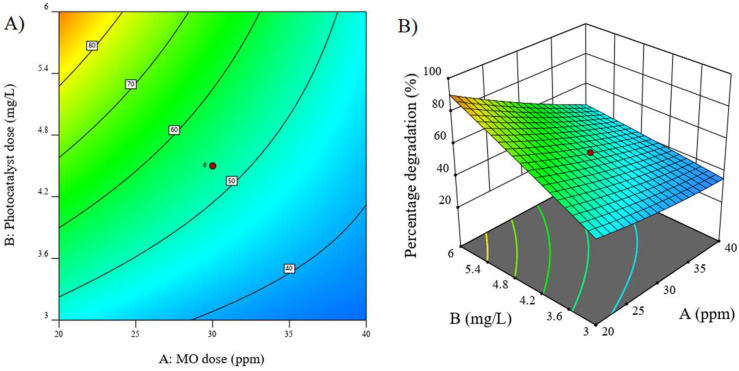
(**A**) Contour plot and (**B**) RSM-based surface plots for the interaction term of AB (MO dose × Photocatalyst dose.

The interaction terms can be better explained on the basis of their contour or three-dimensional surface plots. Figure 10A,B explain the contour and three-dimensional surface plot observed in the case of the interaction factor of AB. Other interaction factors (AC and BC) were found to be statistically insignificant, indicating that the variation in these factor does not affect the D% values of the reaction and will therefore not be discussed here. It is a typical convention to only discus the statistically significant interaction factors via contour or surface plots [12]. As indicated by both these graphs, the maximum degradation indicated by the red colored region was observed at the high CeO_2_-NRs of about 6 mg/L. This is justified as the increment in the concentration of photocatalyst ultimately increased the number of adsorption sites available for the dye molecules to get attached. The adsorbed dye molecule is then able to interact with the free radicals generated in the medium owing to the activation of the photocatalyst (generation of electron–hole pair) by UV light irradiation. These interactions will result in the degradation of the MO dye into less harmful products. As increasing the photocatalyst concentration positively impacts the D% values, higher values of catalyst should be preferred for achieving maximum degradation. Moreover, the lowest MO dose was found to be effective for achieving the maximum degradation. This is again expected as the lower the concentration of MO, the easier it will be to degrade it. There will be no diffusion barrier and the dye molecules will easily interact with the NMs and ultimately be degraded [39].

#### 3.6.5. Optimization

**Figure 11:**
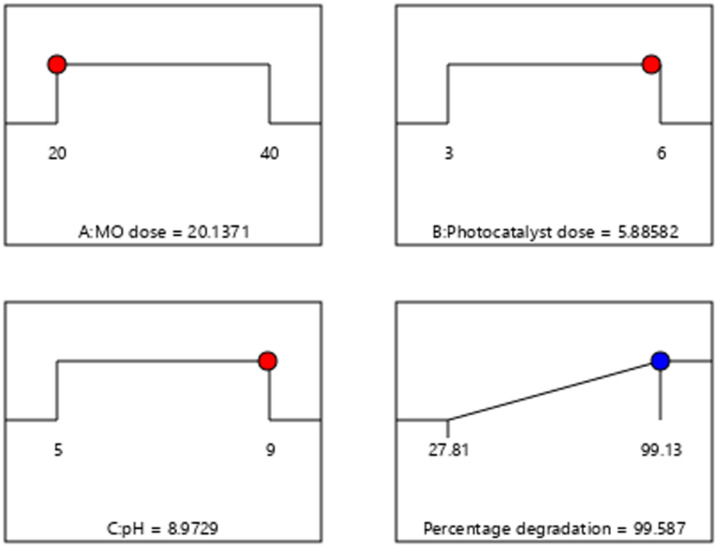
Ramp graph showing the maximum D% values as indicated by the RSM studies.

The RSM-based optimization approach revealed that the maximum degradation value of 99.58% should be achieved with the optimized reaction parameter values of MO dose = 20.1371 ppm; photocatalyst dose = 5.88582 mg/L, and pH units of 8.9729. The results documented by Kaur et al. [40] (optimum pH of 9.00 units), Sithole et al. [41] (optimum MO dose of 20 ppm), and Khalilian et al. [42] (optimum photocatalyst dose of 7 mg/L) relate strongly with the acquired results in our experiment. In order to further validate the optimized parameters provided by the RSM, the typical photocatalytic reaction operating under the specified optimization parameters was carried out. A D% value of 99.317% was observed by using this point prediction analysis, indicating that the model was successful in explaining this model. The ramp graph showing the predictive results at the optimized conditions is exhibited in Figure 11.

## 4. Comparison with the Literature

The acquired results regarding the photocatalytic degradation of MO performed in the presence of the CeO_2_-NRs are presented in Table 3.

## 5. Conclusions

In this study, the phytochemicals present in the *D. sissoo* extract were utilized to engineer CeO_2_-NRs. Not only was this technique found to be effective for the preparation of morphology-specific NMs (i.e., rod-like CeO_2_-NRs), but the NMs were also prepared at rather lower temperatures as opposed to other methodologies. The acquired CeO_2_-NRs were characterized by using XRD, UV-VIS, FTIR, and SEM techniques. All these techniques validated the formation of the NRs by using this phytochemistry-based approach. The CeO_2_-NRs exhibited excellent antioxidant, cytotoxic, and photocatalytic results, indicating that the engineered NRs can be effectively utilized to perform a multitude of practical applications. The best results were found in the case of the antioxidant activity, as the CeO_2_-NRs expressed better antioxidant activity in comparison to industrial antioxidant of BHA. The RSM-based photocatalytic degradation of MO carried out by using the CeO_2_-NRs exhibited the percentage degradation value of 99.317%, which is rather higher in comparison to the degradation percentages documented in the academic literature for the CeO_2_-NRs. Our study signifies that the synthesized CeO_2_-NRs exhibit copacetic potential for multiple applications.

## Figures and Tables

**Table 1:** FRAP, LPO, and CAT assay for the human lymphocytes incubated with CeO_2_-NRs.

$\mathrm{Sample}\;{\mathrm{CeO}}_{2}-\mathrm{NRs}\;(\mu \mathrm{mol}/\mathrm{mL})$	FRAP (μmol/mL)	LPO (μmol/mL)	CAT (units/mL)
15	0.15 ± 0.02	1.89 ± 0.03	1.12 ± 0.01
30	0.23 ± 0.01	1.52 ± 0.01	1.32 ± 0.02
60	0.39 ± 0.01	1.25 ± 0.01	2.20 ± 0.01
120	0.93 ± 0.02	1.85 ± 0.01	2.76 ± 0.01

**Table 2:** The fit statistics and ANOVA analysis for the photocatalytic degradation of MO carried out in the presence of the CeO_2_-NRs.

ANOVA						
Source	Sum of Squares	Degree of Freedom	Mean Square	F-Value	*p*-Value	Degree of Significance
Model	6513.65	9	723.74	149.51	<0.0001	Significant
MO Dose (A)	2564.38	1	2564.38	529.74	<0.0001	
Photocatalyst Dose (B)	2559.89	1	2559.89	528.81	<0.0001	
pH (C)	541.83	1	541.83	111.93	<0.0001	
AB	529.10	1	529.10	109.30	<0.0001	
AC	80.14	1	80.14	16.55	0.0023	
BC	18.91	1	18.91	3.91	0.0763	
A^2^	48.86	1	48.86	10.09	0.0099	
B^2^	2.41	1	2.41	0.4983	0.4964	
C^2^	175.23	1	175.23	36.20	0.0001	
Residual	48.41	10	4.84			
Lack of Fit	47.62	5	9.52	60.50	0.0002	Not Significant
Pure Error	0.7872	5	0.1574			
Cor Total	6562.06	19				
Fit Statistics						
Standard Deviation				2.20		
Square of Regression Coefficient (R^2^)				0.9926		
Adjusted R^2^				0.9860		
Predicted R^2^				0.9429		

**Table 3:** Comparison with the literature.

Photocatalysts	Preparation Method	Irradiation Source	Degradation Percentage	Reference
Ce-ZnO	Hydrothermal	Fluorescent lamp	94.06%	[16]
CeO_2_-NPs	*Calotropis procera* flower extract	Sunlight	98%	[43]
CeO_2_/SrFe_12_O_19_ NCs	Co-precipitation	Xe lamp (UV radiations)	88.38%	[44]
Pd-CeO_2_	Precipitation and impregnation	Halogen lamp	92%	[45]
CeVO_4_/BiVO_4_/rGO NCs	Solvothermal	Xe lamp	98%	[46]
CeO_2_-NRs	*Dalbergia sissoo* extract	UV lamp	99.317%	This study

Abbreviations: Cerium-doped zinc oxide (Ce-ZnO); cerium oxide nanoparticles (CeO_2_-NPs); hexaferrite strontium ferrite (SrFe_12_O_19_); nanocomposites (NCs); Xenon (Xe); palladium-doped cerium oxide (Pd-CeO_2_); bismuth vanadate (BiVO_4_); cerium vanadate (CeVO_4_); reduced graphene oxide (rGO).

## Data Availability

The data will be made available upon request.

## Supplementary Materials

The following supporting information can be downloaded at: https://www.mdpi.com/article/10.3390/molecules27238188/s1, Table S1. Reaction variables utilized for developing 3-factor based Central Composite Design (CCD) for the photocatalytic degradation of methyl orange (MO) in the presence of CeO2-NRs. Table S2. CCD based design of experiment for the photocatalytic degradation reaction of MO. Table S3. Statistical analysis for investigating the best predictive model for the photocatalytic degradation of MO performed in the presence of CeO2-NRs.
