# Supplementary material for: Cerium Oxide Nanorods Synthesized by Dalbergia sissoo Extract for Antioxidant, Cytotoxicity, and Photocatalytic Applications
Source: Molecules. 2022 Nov 24;27(23):8188. doi: 10.3390/molecules27238188 (PMC9735491; doi:10.3390/molecules27238188)
Supplement: Supplementary file 1 [file molecules-27-08188-s001.zip › molecules-2028973-SI.pdf]

Supplementary information

Cerium Oxide Nanorods Synthesized by *Dalbergia sissoo* Extract for Antioxidant, Cytotoxicity, and Photocatalytic Applications

Mir Waqas Alam [1,2,*], Sumaira Naeem [3,*], Sheikh Muhammad Usman [4], Qudsia Kanwal [5], Amal BaQais [6], Fatimah Saeed Aldughaylibi [1,2], Insha Nahvi [1,7] and Noushi Zaidi [1,7]

[1] Al Bilad Bank Scholarly Chair for Food Security in Saudi Arabia, The Deanship of Scientific Research, The vice Presidency for Graduate Studies and Scientific Research, King Faisal University, Al-Ahsa 31982, Saudi Arabia

[2] Department of Physics, College of Science, King Faisal University, Al-Ahsa 31982, Saudi Arabia

[3] Department of Chemistry, University of Gujrat, Gujrat 50700, Pakistan

[4] Hunza Sugar Mills Private Limited (Distillery Division), Lahore 54000, Pakistan

[5] Department of Chemistry, The University of Lahore, Lahore 54000, Pakistan

[6] Department of Chemistry, College of Science, Princess Nourah Bint Abdulrahman University, Riyadh 11671, Saudi Arabia

[7] Department of Basic Sciences, Preparatory Year Deanship, King Faisal University, Al-Ahsa 31982, Saudi Arabia

1. RSM studies

1.1. Variables, units and ranges

Table S1. Reaction variables utilized for developing 3-factor based Central Composite Design (CCD) for the photocatalytic degradation of methyl orange (MO) in the presence of CeO_2-NRs.

Reaction variables	Units	Codes	Low (-1)	High (+1)	Minimum value	Maximum value	Mean
MO dose	Ppm	A	20.00	40.00	13.18	46.82	30.00
Photocatalyst dose	mg/mL	B	3.00	6.00	1.98	7.02	4.50
pH	unit	C	5.00	9.00	3.64	10.36	7.00

1.2. The 3-factor CCD based standard table

The standard design of experiment acquired by using 3-factor CCD matrix is presented in table S2. The acquired experimental order was not followed but in fact the experiments were performed randomly in order to reduce the unforeseen errors in the experiments

Table S2. CCD based design of experiment for the photocatalytic degradation reaction of MO.

Standard Order	Experimental run order	MO dose (A) (ppm)	Photocatalyst dose (B) (mg)	pH values (C) (units)	Response (%D) Experimental values	Predicted response (%D) with respect to Quadratic model
20	1	30	4.5	7	53.01	53.29
9	2	13.1821	4.5	7	83.96	81.54
17	3	30	4.5	7	53.83	53.29
2	4	40	3	5	35.54	34.27
16	5	30	4.5	7	52.87	53.29
10	6	46.8179	4.5	7	34.81	35.45
3	7	20	6	5	83.95	85.81
4	8	40	6	5	45.95	48.46
13	9	30	4.5	3.63641	54.96	52.56
8	10	40	6	9	52.36	51.66

18	11	30	4.5	7	52.91	53.29
6	12	40	3	9	44.21	43.61
12	13	30	7.02269	7	78.23	75.15
15	14	30	4.5	7	53.18	53.29
1	15	20	3	5	37.12	39.08
7	16	20	6	9	99.13	100.00
14	17	30	4.5	10.3636	73.12	73.74
11	18	30	1.97731	7	27.81	29.10
19	19	30	4.5	7	53.61	53.29
5	20	20	3	9	62.34	61.09

1.3. Model selection

The complete details regarding the statistics applied for the model selection is provided in the table S3.

Table S3. Statistical analysis for investigating the best predictive model for the photocatalytic degradation of MO performed in the presence of CeO_2-NRs.

Model Fit Summary								
**Source**	**Standard deviation**	**Sequential p-value**	**Lack of Fit p-value**	**R^2**	**Adjusted R^2**	**Predicted R^2**	**PRESS**	**Remarks**
Linear	7.48	< 0.0001	< 0.0001	0.8635	0.8379	0.7539	1615.01	Not suggested
2-factor interaction (2FI)	4.54	0.0010	< 0.0001	0.9592	0.9404	0.9078	605.28	Not suggested
**Quadratic**	**2.20**	**0.0005**	**0.0002**	**0.9926**	**0.9860**	**0.9429**	**374.41**	**Suggested**
Cubic	1.21	0.0204	0.0009	0.9987	0.9958	0.7325	1755.25	Aliased

Sequential Model Sum of Squares [Type-I]						
**Source**	**Sum of Squares**	**Degrees of freedom**	**Mean Square**	**F-value**	**p-value**	**Remarks**
Mean vs Total	64173.12	1	64173.12			--
Linear vs mean	5666.10	3	1888.70	33.73	< 0.0001	Not suggested
2FI vs Linear	628.15	3	209.38	10.16	0.0010	Not suggested
**Quadratic vs 2FI**	**219.40**	**3**	**73.13**	**15.11**	**0.0005**	**Suggested**
Cubic vs Quadratic	39.66	4	9.92	6.80	0.0204	Aliased
Residual	8.74	6	1.46			--
Total	70735.18	20	3536.76			--

Lack of fit tests						
**Source**	**Sum of Squares**	**Degrees of freedom**	**Mean Square**	**F-value**	**p-value**	**Remarks**
Linear	895.17	11	81.38	516.92	< 0.0001	Not Suggested
2FI	267.02	8	33.38	212.01	< 0.0001	Not Suggested
**Quadratic**	**47.62**	**5**	**9.52**	**60.50**	**0.0002**	**Suggested**
Cubic	7.96	1	7.96	50.55	0.0009	Aliased
Pure Error	0.7872	5	0.1574			
